# Effects of combined nitrogen and phosphorus application on protein fractions and nonstructural carbohydrate of alfalfa

**DOI:** 10.3389/fpls.2023.1124664

**Published:** 2023-03-08

**Authors:** Jiantao Zhao, Rongzheng Huang, Xuzhe Wang, Chunhui Ma, Man Li, Qianbing Zhang

**Affiliations:** ^1^ College of Animal Science and Technology, Shihezi University, Shihezi, China; ^2^ College of Medicine, Shihezi University, Shihezi, China

**Keywords:** alfalfa, nitrogen and phosphorus combined, protein fractions, nonstructural carbohydrate, hay yield

## Abstract

Nitrogen (N) and phosphorus (P) fertilization significantly affect alfalfa production and chemical composition; however, the effect of combined N and P application on protein fractions and the nonstructural carbohydrate content of alfalfa is not fully understood. This two-year study investigated the effects of N and P fertilization on the protein fractions, nonstructural carbohydrates (NSC), and alfalfa hay yield. Field experiments were carried out using two nitrogen application rates (N60, 60 and N120, 120 kg N ha*^−^* ^1^) and four phosphorus application rates (P0, 0; P50, 50; P100, 100; and P150, 150 kg P ha*^−^* ^1^), total 8 treatment (N60P0, N60P50, N60P100, N60P150, N120P0, N120P50, N120P100 and N120P150). Alfalfa seeds were sown in the spring of 2019, uniformly managed for alfalfa establishment, and tested in the spring of 2021–2022. Results indicated that P fertilization significantly increased the hay yield (3.07–13.43% ranges), crude protein (6.79–9.54%), non-protein nitrogen of crude protein (fraction A) (4.09–6.40%), and NSC content (11.00–19.40%) of alfalfa under the same treatment of N application (*p* < 0.05), whereas non-degradable protein (fraction C) decreased significantly (6.85–13.30%, *p* < 0.05). Moreover, increasing N application resulted in a linear increase the content of non-protein N (NPN) (4.56–14.09%), soluble protein (SOLP) (3.48–9.70%), and neutral detergent-insoluble protein (NDIP) (2.75–5.89%) (*p* < 0.05), whereas acid detergent-insoluble protein (ADIP) content was significantly decreased (0.56–5.06%, *p* < 0.05). The regression equations for nitrogen and phosphorus application indicated a quadratic relationship between yield and forage nutritive values. Meanwhile, the comprehensive evaluation scores of NSC, nitrogen distribution, protein fractions, and hay yield by principal component analysis (PCA) revealed that the N120P100 treatment had the highest score. Overall, 120 kg N ha*^−^* ^1^ coupled with 100 kg P ha*^−^* ^1^ (N120P100) promoted the growth and development of perennial alfalfa, increased soluble nitrogen compounds and total carbohydrate content, and reduced protein degradation, thus improving the alfalfa hay yield and nutritional quality.

## 1 Introduction

Due to the increasing population, forage quality and yield issues are becoming increasingly important for animal production. Alfalfa is widely planted as high-quality forage because of its good palatability, high crude protein content, and high hay yield (Yuan et al., 2022). In recent years, with the development of large-scale and standardized animal husbandry, the demand for high-quality and yield forage has increased year by year, which has led to an intense increase in the demand for alfalfa. The shortage of high-quality roughage and the quantity of imported alfalfa hay cannot meet the needs of the husbandry, and the situation is getting more and more serious. A low nutritional value of forage could be due to poor soil fertility and the absence or low level of fertilization (Alencar et al., 2018). Xinjiang is in an arid to semi-arid area of the Asian, with little rainfall all year round, an arid climate, and strong evaporation. Alfalfa is widely planted in the region because of its strong nitrogen fixation ability and high drought and cold resistance. Nitrogen (N) and phosphorus (P) play important roles in plant function, which is affected by limiting nutrients in ecosystems (Luo et al., 2013). Previous studies have observed that the improved nutritional quality of forage grasses is due to the fact that N fertilization enhanced crude protein and reduced crude fiber contents (Sun et al., 2022). P is an essential and abundant element that plays a vital role in plant growth and development. It was found that adding P fertilizer improved the quality and yield of alfalfa through three consecutive years of field trials (Zhang et al., 2020a). Studies have shown that the quality and yield of alfalfa are more significantly affected by the combination of N and P than by single N or P fertilization (Gu et al., 2022). However, excessive P application under N-deficient conditions might not be beneficial. Previous studies on the inter­play between N and P emphasized a reduction in forage yield induced by N deficit and the reciprocal impact of P deficiency on forage quality (Zhang et al., 2021). Therefore, the balanced application of N and P is important for improving forage yield and quality.

In addition to high yields, excellent forage nutritive value is the most desirable goal because of its direct impact on the profitability of forage production and the livestock enterprises it supports. In forages, the concentration of protein, starch and soluble sugars are essential because, when consumed by the animals, these are supplied to the rumen microbes and therefore affect animal maintenance and production. The nonstructural carbohydrates (NSC), which play different roles in plant energy metabolism, transportation, and storage carbohydrates, are mainly starch and soluble sugars (Hartmann and Trumbore, 2016). The NSC content not only reflects the balance between plant carbon uptake (photosynthetic assimilation) and carbon consumption (respiration and growth) but also indirectly indicates the growth and nutritional value of the plant. Studies on the effects of N fertilizer addition on changes in NSC content have not yet reached a definite conclusion. It was found that the content of starch, soluble sugars, and NSC in *Reaumuria soongorica* leaves was increased by N fertilization (Zhang et al., 2020b). However, it was also found that N fertilizer addition reduces the NSC content of maize leaves (Wu et al., 2020). However, adequate information regarding the nutritional value of grasses in terms to improve the profile of protein fractions with fertilization is lacking. Given the high cost of chemical fertilization and the pollution of agricultural soils by excessive application, it is critical to determine the best fertilization strategy to improve yields and quality and reduce the cost of alfalfa production in semi-arid regions.

Protein and carbohydrates are the most important nutrients in determining animal performance, and their content and availability vary with fertilization levels and management practices. The Cornell Net Carbohydrate and Protein System (CNCPS) is increasingly used to assess the nutritional value of feeds, enabling an accurate representation of the nutrient content of feeds based on the solubility of each fraction in the rumen. CNCPS accurately splits the nutrient content of feed and accurately predicts the nutritional requirements and production performance of ruminants. Reduced animal performance is partly due to different fertilization levels and strategies to reduce the concentration and availability of protein in the forage. Previous studies have observed that the improved nutritional quality of forage grasses is due to N fertilization increasing nitrogen compounds (non-protein nitrogen (NPN) and soluble protein (SOLP)) (Berca et al., 2021). It has been found that applying N fertilization can improve the quality of forage by increasing the total degradable and true protein contents (Leite et al., 2021). However, adequate information regarding the nutritional value of alfalfa in terms of improving the profile of protein fractions by using different combined N and P application strategies is lacking. Therefore, the purpose of this study is to find the optimal ratio of nitrogen and phosphorus to improve the quality and yield of alfalfa high-quality forage, and thus improve the livestock productivity. The present study hypothesized that the concentrations of non-protein nitrogen of crude protein (fraction A) would increase, and the starch and soluble sugar contents would increase with N and P fertilization. Therefore, to test this hypothesis, we conducted a 2-year field trial. The hay yield was used to evaluate plant growth, the starch and soluble sugar contents were used to study the dynamic changes in NSC, and the fraction A and ruminal degradable protein (fraction B) contents were used to study the dynamic changes in CNCPS and to evaluate the quality of alfalfa.

## 2 Materials and methods

### 2.1 Experimental site description

The field experiments were conducted in 2021 and 2022 at the Water-saving Irrigation Experiment Station of Shihezi University, Shihezi (44°20′ N, 88°30′ E, 450.8 m above sea level), located in Xinjiang Province, Northwest China. The climate is temperate continental, dry and with little rain. The average annual temperature is 7°C, the frost-free period is 168~171 d, the annual precipitation is 190~260 mm, the annual evaporation is 1000~1500 mm and the average annual sunshine time is 2770 h. Soil properties measured before the experiment were as follows; total nitrogen 1.18 g kg^−1^, alkaline nitrogen 145.47 mg·kg^−1^, total phosphorus 0.53 g·kg^−1^, available phosphorus 19.30 mg·kg^−1^, potassium 119.8 mg·kg^−1^, density 1.54 g·cm^−3^, and organic matter 39.5 g·kg^−1^.

### 2.2 Experimental design and treatments management

Alfalfa stand was established in 2019, uniformly managed for alfalfa establishment, and they were tested in spring 2021–2022, with a seeding drill at rate of 18 kg·ha^−1^, the row spacing of 20 cm, the sowing depth was 2.0 cm and a plot area of 24 m^2^ (4 m×6 m). In this way, the experiment comprised of eight treatments, and each was replicated three times in a two-factor completely randomized design. The N application rates were 60 (N60) and 120 (N120) kg*·*ha*^−^* ^1^ (as urea equivalent), and the P application rates included 0 (P0), 50 (P50), 100 (P100) and 150 (P150) kg·ha*^−^* ^1^ (as P_2_O_5_ equivalent). The N was added as urea (46% total N) and P was added as monoammonium phosphate (with a low amount of N) (52% P_2_O_5_, 12.2% total N, respectively), because they are the commonly used fertilizers in the study area. After P fertilizer was applied, the imbalance in N treatments was balanced by the additional applications of N fertilizer to maintain the same N application rate in the different P treatments. The fertilizer was added to the fertilizer tank and applied together with water at the green-up stage (19 April 2021 and 25 April 2022) and 3−5 d after the first, second and third clippings (27 May, 3 July, 4 August, 2021, 30 May, 4 July, 3 August 2022).

### 2.3 Plant material and growth measurements

Alfalfa was harvested at the early flowering stage (10% of blooming), four times per year. However, this trial involved only the first clipping of alfalfa and was harvested on May 27 and 30 in 2021 and 2022, respectively. At harvest, three representative sample squares (1 m × 1 m), cut approximately 5 cm high, were randomly selected in the center of each plot and used to determine alfalfa yield. The samples were oven dried at 105°C for 30 min and then oven dried at 65°C to a constant weight for determining the dry biomass of fodder. Hay yield was determined using dry matter (Fan et al., 2016). Dried plant samples were ground into fine powder to pass through a 0.04 in screen and used for nutritive analyses.

### 2.4 Nutritional quality

Weigh 0.5g of the crushed and sieved dried sample into a digestion tube, add concentrated sulphuric acid and catalyst, place a small funnel over the mouth of the bottle and place it on the digestion oven. After digestion, the digest was transferred without damage to a 100 ML volumetric flask, the volume was set and cooled, and 10 ML of liquid was taken. The N contents were determined by the automatic Kjeldahl nitrogen analyzer (K9840, Hanon Co., Ltd., Qingdao, China) and crude protein contents (CP) was estimated as N% × 6.25. Neutral detergent fiber (NDF) and acid detergent fiber (ADF) contents were analyzed by the VanSoest et al. (1991) method. Weigh 0.5g of the sample into a fibrous bag and seal it, boil it for 60 minutes in a neutral detergent or acid detergent, soak it in acetone for 30 minutes, wash the bag with water and put it in an oven (60 °) until it reaches a constant weight.

### 2.5 Analysis for protein fractions (CNCPS)

Soluble protein (SOLP) concentrations were analyzed according to Bradford (1976), some modifications were made to determine the SOLP content. Take 43.5 g of Na_2_HPO_4_ · 12H_2_O (71.64 g L) and 56.5 g of NaH_2_PO_4_ · 2H_2_O (31.2 g L), fix the volume to 1 L, and configure it into Borate-phosphate buffer solution (pH=6.7-6.8), weigh 0.05 g of the ground and dried sample, and place it in a 15 ml centrifuge tube. Add 10 ml Borate-phosphate buffer solution and 1 ml sodium azide solution. After standing for 3 h (20-25°C), the sample was centrifuged at 4000 r min^-1^ for 10 min. The absorbance of the solutions of SOLP after reaction with Coomassie brilliant blue G-250 reagent was measured at 595 nm using a spectrophotometer. The NPN, neutral detergent-insoluble protein (NDIP), and acid detergent-insoluble protein (ADIP) concentrations were analyzed according to Licitra et al. (1996). Determination of NDIP and ADIP: the samples of neutral detergent fiber and acid detergent fiber were dried and digested to determine the residual nitrogen. Determination of NPN: Weigh 0.5 g of sample into a 250 ml conical flask, add 50 ml of distilled water and let stand for 30 min. Add 10 ml of 10% trichloroacetic acid and allow to stand for 30 min. Filter and wash twice with trichloroacetic acid solution. The filter paper was dried and digested to determine the residual nitrogen. The determination of NDIP and ADIP is to digest the samples dried after measuring the medium wash and acid wash and determine the residual nitrogen. The protein fraction were analyzed according to the Cornell Net Carbohydrate and Protein System (CNCPS) (Sniffen et al., 1992; Licitra et al., 1996). The CP content was divided into instantaneously solubilizable protein A (PA), completely undegradable (PC) and potentially degradable true protein (PB). The PB was further sub-divided into rapidly (PB_1_), intermediately (PB_2_), and slowly (PB_3_) degradable true protein. The calculation formula is as follows: PA (% CP) = NPN (%SOLP) × 0.01 × SOLP (% CP); PB_1_ = SOLP (% CP)-PA (% CP); PB_2_ = 100-PA (% CP)-PB_1_ (% CP)-PB_3_ (% CP)-PC (% CP); PB_3_ = NDIP (% CP)-ADIP (% CP); PC = ADIP (% CP).

### 2.6 Non-structural carbohydrate determination

NSC concentration was defined as the sum of soluble sugar and starch content. The soluble sugar and starch content were analyzed according to Hajihashemi et al. (2020) and Wang et al. (2021). Precisely weigh 0.2 g of the sample and place it in a 15 ml centrifuge tube. Add 4 ML of 80% ethanol solution to the centrifuge tube and leave it in a water bath at 80°C for 30 minutes. The solution was cooled to room temperature and then centrifuged at 4000 r min^-1^ for 10 min. The extraction process was repeated 3 times. The supernatant was retained for the determination of soluble sugar content. To the precipitate, 2 mL of distilled water was added and boiled for 15 min. After the solution was cooled to room temperature, 3 mL of 9.2 M HClO_4_ solution was added and shaken for 15 min. 4 mL of distilled water was added and centrifuged at 4800 r min^-1^ for 10 min. The supernatant was retained and further extracted with 3 mL of 4.6 M HClO_4_. All supernatants were collected for determination of amylose content.

### 2.7 Statistical analysis

Figures were constructed with Excel 2016 (Microsoft Corp., USA) and Origin 2021 (Origin Corp., USA). Analysis of variance (ANOVA) for the two-year data was performed using the SPSS 20.0 statistical (IBM Corp., USA). Repeated-measures analysis of variance was used to determine the effects of N and P on agronomic traits, protein fractions, nonstructural carbohydrate and hay yield of alfalfa. Tukey’s significant difference test was used to compare the significant differences among the treatment means, and interaction effects, wherever found significant were also calculated and presented. The relationships of forage yield and qualitative indexes were checked for normality in relation to treatments in different years using Origin 2021, and linear and nonlinear regression analyses were per formed.

#### 2.3.1 Mathematical model for comprehensive evaluation by principal component analysis:

##### 2.3.1.1 Normalizing raw data


$${\tilde{X}}_{\text{ij}}=\frac{X_{\text{ij}}-{\tilde{X}}_{\text{j}}}{S_{\text{j}}},\left(i=1,2,\ldots ,n;j=1,2,\ldots ,m\right)$$

##### 2.3.1.2 Establish the correlation coefficient matrix R between the variables

Related Matrix *R*=(*r*_*ij*_)_*m*×*m*_


$$r_{i\;j}=\frac{\sum_{k=1}^{\text{n}}{\tilde{X}}_{ki}\cdot {\tilde{X}}_{kj}}{n-1},(i,j=1,2,\ldots ,m)$$

##### 2.3.1.3 Calculate the eigenvalues and eigenvectors of the correlation coefficient matrix R


$$\left\{ \begin{array}{l} y_{1}=u_{11}{\tilde{x}}_{1}\;+\;u_{21}{\tilde{x}}_{2}\;+\;\ldots +\;u_{n1}{\tilde{x}}_{n}\\ y_{2}=u_{12}{\tilde{x}}_{1}\;+\;u_{22}{\tilde{x}}_{2}\;+\;\ldots +\;u_{n2}{\tilde{x}}_{n}\\ \ldots \ldots \ldots \ldots \ldots ⇕\ldots \ldots \ldots \ldots \ldots \\ y_{m}=u_{1m}{\tilde{x}}_{1}\;+\;u_{2m}{\tilde{x}}_{2}\;+\;\ldots +\;u_{nm}{\tilde{x}}_{n} \end{array}\right.$$

##### 2.3.1.4 Calculate the composite score

Calculate the information contribution rate and cumulative contribution rate of the given value *λ*_j_(*j*=1,2,...,*m*)

The information contribution of the principal components is $b_{\text{j}}=\frac{\lambda_{\text{j}}}{\sum_{k=1}^{m}\lambda_{k}}(j=1,2,\ldots ,m)$

The cumulative contribution of the principal components is $\alpha_{\text{p}}=\frac{\sum_{k=1}^{p}\lambda_{k}}{\sum_{k=1}^{m}\lambda_{k}}$

Calculate the overall score $Z=\sum_{j=1}^{p}b_{j}y_{j}(j=1,2,\ldots ,m)$

## 3 Results

### 3.1 Effects of nitrogen and phosphorus combined application on nitrogen distribution

**Figure 1:**
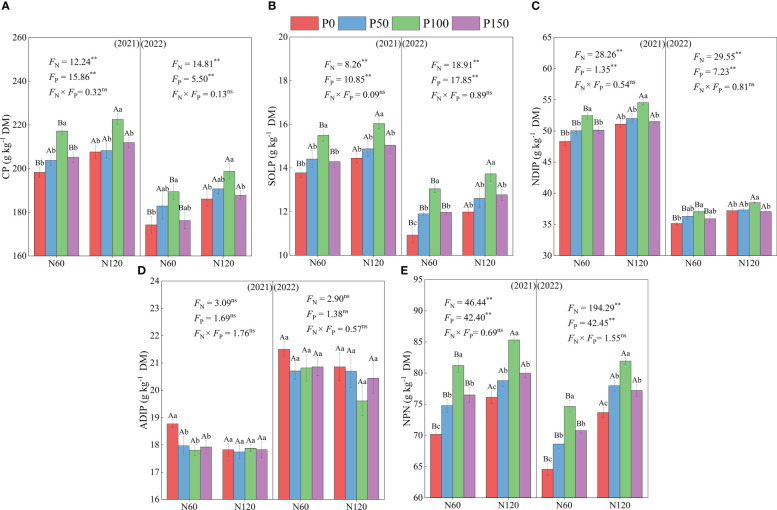
CP, crude protein **(A)**, SOLP, soluble protein **(B)**, NDIP, neutral detergent-insoluble protein **(C)**, ADIP, acid detergent-insoluble protein **(D)** and NPN, non-protein nitrogen **(E)** of alfalfa during difference nitrogen and phosphorus combined fertilization in 2021 to 2022. All the values were the means of four replicates with standard errors. Different small letters indicate significant differences between different P fertilizer treatments under the same N application condition (*p* < 0.05). Different capital letters indicate significant differences between different N fertilizer levels under the same P application condition (*p* < 0.05). F_N_, F_P_ and F_N_ × F_P_ represent the F value under the N application levels, P application levels and the interaction of N and P application levels, respectively. ns indicates no significant difference (*p* > 0.05), * indicates significant difference (*p* < 0.05) and ** indicates extremely significant difference (*p* < 0.01).

Nitrogen (N) and phosphorus (P) fertilization showed significant effects (*p* < 0.05) on crude protein (CP), soluble protein (SOLP), neutral detergent-insoluble protein (NDIP) and non-protein nitrogen (NPN) content of alfalfa. The CP, SOLP, NDIP and NPN contents showed an increasing and then decreasing trend, while the ADIP content showed a decreasing and then increasing trend with the increase of P application under the same N treatment from 2021 to 2022 (**Figure 1**). Meanwhile, the CP, SOLP, NDIP and NPN contents were significantly greater in the P100 treatment than P0 treatments (in the 6.79 – 9.54, 11.00 – 19.40, 3.46 – 8.54, and 11.18 – 15.73% ranges, respectively, *p* < 0.05), while the ADIP contents were significantly lower than in the P0 treatment in 2021 (in the 0.22 – 5.99% ranges, *p* < 0.05). In addition, CP, SOLP, NDIP and NPN contents were significantly different under each P treatment (*p* < 0.05), and ADIP contents were not significantly different (*p* > 0.05). Under the same P treatment, the CP, SOLP, NDIP and NPN contents showed an increasing trend with the increase of N fertilization (in the 2.50 – 6.81, 3.48 – 9.70, 2.75 – 5.89, and 4.56 – 14.09% ranges, respectively, *p* < 0.05), while the ADIP content showed a decreasing trend (in the 0.56 –5.06% range, *p* > 0.05). In addition, CP, SOLP, NDIP and NPN contents were significantly different (*p* < 0.05), and ADIP contents were not significant (*p* > 0.05) under N120 treatment than N100 treatment from 2021 to 2022.

### 3.2 Effects of nitrogen and phosphorus combined application on protein fractions

**Figure 2:**
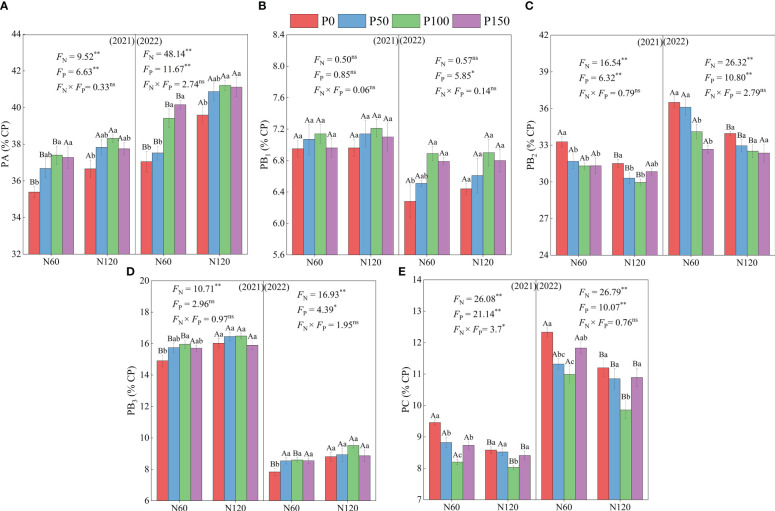
Effect of nitrogen and phosphorus combined fertilization on protein fraction of PA = non-protein nitrogen of crude protein **(A)**, PB_1_ = easily degradable protein **(B)**, PB_2_ = intermediately degradable protein **(C)**, PB_3_ = slowly degradable protein **(D)**, and PC = non-degradable protein **(E)** of alfalfa. Different small letters indicate significant differences between different P fertilizer treatments under the same N application condition (*p* < 0.05). Different capital letters indicate significant differences between different N fertilizer levels under the same P application condition (*p* < 0.05). F_N_, F_P_ and F_N_ × F_P_ represent the F value under the N application levels, P application levels and the interaction of N and P application levels, respectively. ns indicates no significant difference (*p* > 0.05), * indicates significant difference (*p* < 0.05) and ** indicates extremely significant difference (*p* < 0.01).

The contents of PA, PB_1_, and PB_3_ in the protein fractions showed an increasing trend and then decreased with an increase in P fertilization, while the contents of PB_2_ and PC showed a trend of decreasing and then increasing P application (**Figure 2**). Under N60 conditions, PA and PB_3_ contents were significantly increased (4.09–6.40 and 2.81–9.69%, respectively, *p* < 0.05), and PB_2_ and PC contents were significantly lower (4.23–6.61% and 6.85–13.30%, respectively, *p* < 0.05) under P100 treatment compared to P0 treatment in 2021 and 2022. Moreover, the PB_1_ content was significantly different (*p* < 0.05) after P100 treatment in 2022. Under the same P application treatment, PA and PB_3_ contents were significantly greater under the N120 treatment than under the N60 treatment (2.37–8.93 and 2.81–9.69%, respectively, *p* < 0.05), while PB_2_ and PC contents were significantly lower than the N60 treatment (1.53–6.98 and 2.07–10.28%, respectively, *p* < 0.05). In addition, PA, PB_2_, PB_3_, and PC contents differed significantly (*p* < 0.05), whereas PB_1_ content did not differ significantly between the N treatments in 2021 and 2022 (*p* > 0.05).

### 3.3 Effects of nitrogen and phosphorus combined application on non-structural carbohydrate

**Figure 3:**
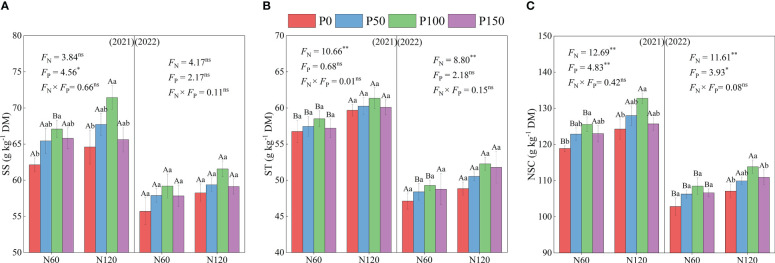
SS, soluble sugar **(A)**, ST, starch **(B)** and NSC, non-structural carbohydrate **(C)** of alfalfa during difference nitrogen and phosphorus combined fertilization in 2021 to 2022. Different small letters indicate significant differences between different P fertilizer treatments under the same N application condition (*p* < 0.05). Different capital letters indicate significant differences between different N fertilizer levels under the same P application condition (*p* < 0.05). F_N_, F_P_ and F_N_ × F_P_ represent the F value under the N application levels, P application levels and the interaction of N and P application levels, respectively. ns indicates no significant difference (*p* > 0.05), * indicates significant difference (*p* < 0.05) and ** indicates extremely significant difference (*p* < 0.01).

The soluble sugar (SS), starch (ST), and nonstructural carbohydrate (NSC) contents showed a trend of increasing and then decreasing with an increase in P fertilization under the same N fertilization treatment (**Figure 3**). Meanwhile, the SS and NSC content of the P100 treatment was significantly higher than that of the P0 treatment (5.68–10.61 and 11.00–19.40%, respectively, *p* < 0.05), while the difference in starch was not significant in 2021 (*p* > 0.05). In addition, the difference in NSC between the P treatments was significant (*p* < 0.05) in 2021 and 2022, and soluble sugar content was significant (*p* < 0.05) only in 2021. Under the same P treatment, the SS, ST, and NSC content increased with increasing N application, and ST and NSC content was significantly higher in N120 than in the N60 treatment (2.23–6.75, 3.65–6.17, and 3.48–9.70%, respectively, *p* < 0.05), while soluble sugar content showed no significant difference (*p* > 0.05).

### 3.4 Effects of nitrogen and phosphorus combined application on hay yield

**Figure 4:**
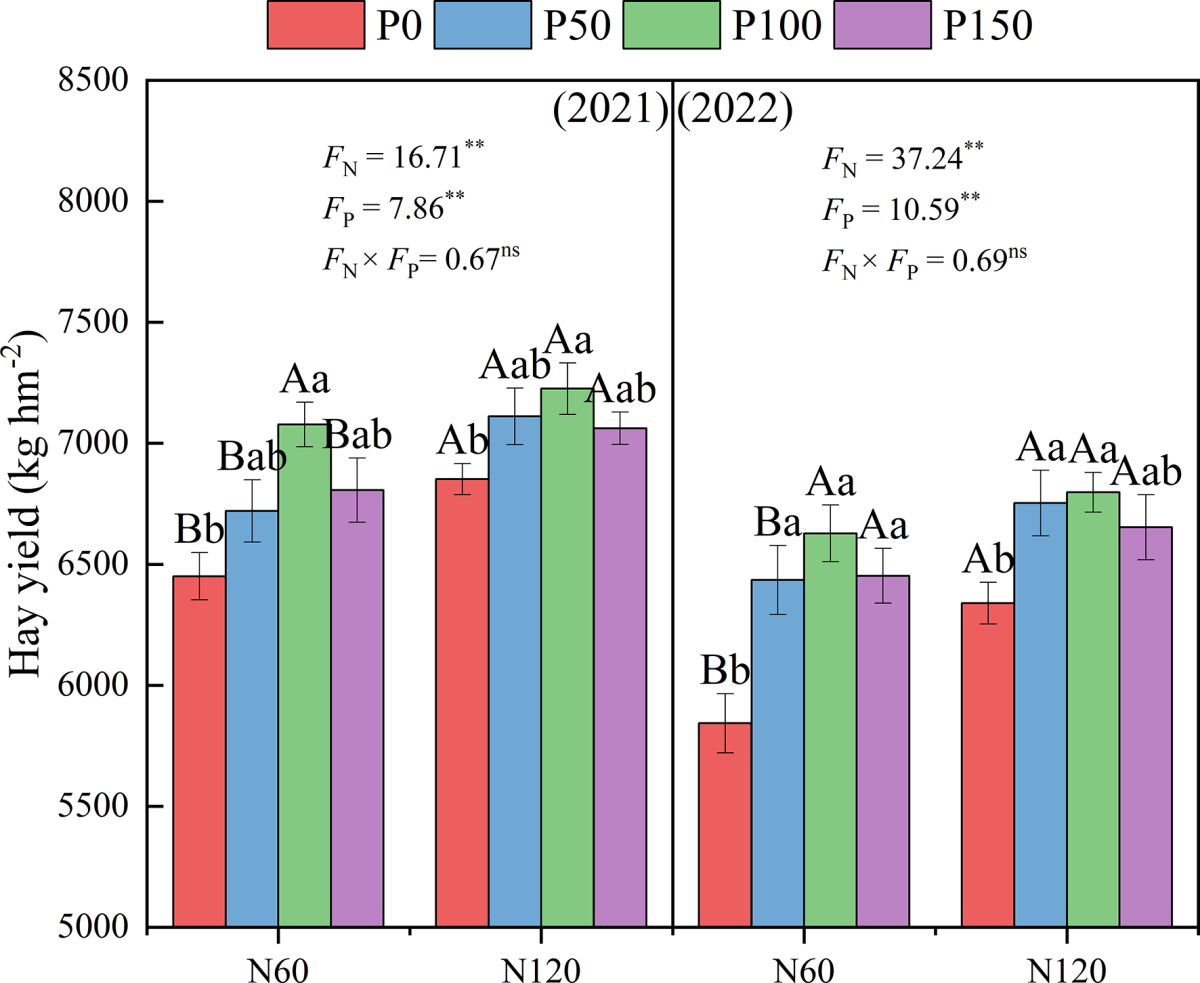
Effect of nitrogen and phosphorus combined fertilization on the hay yield (first clipping) of alfalfa in 2021 to 2022. Different small letters indicate significant differences between different P fertilizer treatments under the same N application condition (*p* < 0.05). Different capital letters indicate significant differences between different N fertilizer levels under the same P application condition (*p* < 0.05). F_N_, F_P_ and F_N_ × F_P_ represent the F value under the N application levels, P application levels and the interaction of N and P application levels, respectively. ns indicates no significant difference (*p* > 0.05), * indicates significant difference (*p* < 0.05) and ** indicates extremely significant difference (*p* < 0.01).

Combined nitrogen and phosphorus fertilization showed significant effects (*p* < 0.05) on the hay yield of alfalfa (**Figure 4**). Under the same N treatment, alfalfa hay yield showed a trend of increasing and then decreasing with increasing P application, reaching the maximum under P100, and was significantly higher than the P0 treatment (3.07–13.43%, *p* < 0.05) in 2021 and 2022. Under the same P treatment, alfalfa hay yield increased with increasing N application, with the N120 treatment being significantly higher than the N60 treatment (2.11–8.49%, *p* < 0.05) in 2021 and 2022.

### 3.5 Relationship of measured indexes and phosphorus under nitrogen fertilization

**Figure 5:**
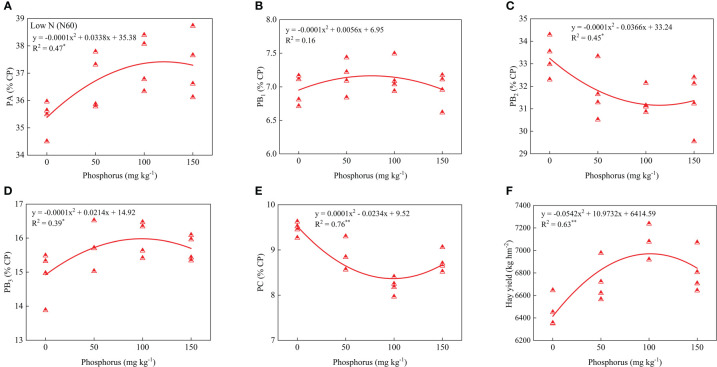
Relationship of PA **(A)**, PB_1_ **(B)**, PB_2_ **(C)**, PB_3_ **(D)**, PC **(E)**, and forage yield **(F)** with phosphorus treatments under low nitrogen condition. * and ** indicates the significant level at *p* ≤ 0.05 and *p* ≤ 0.01, respectively. The X and Y axes were adjusted to minimize the graph’s blank areas and highlight the relationship.

**Figure 6:**
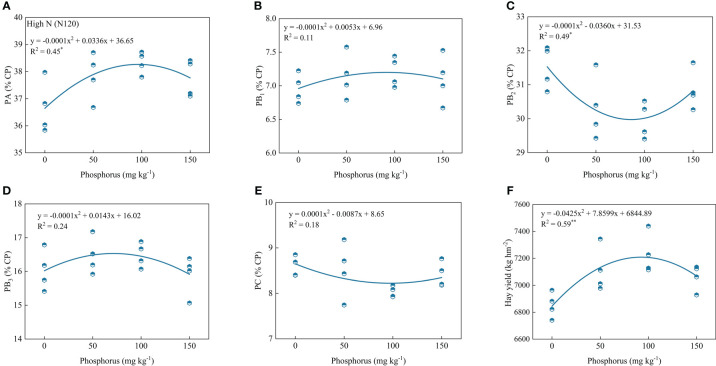
Relationship of PA **(A)**, PB_1_ **(B)**, PB_2_ **(C)**, PB_3_ **(D)**, PC **(E)** and forage yield **(F)** with phosphorus treatments under high nitrogen condition. * and ** indicates the significant level at *p* ≤ 0.05 and *p* ≤ 0.01, respectively. The X and Y axes were adjusted to minimize the graph’s blank areas and highlight the relationship.

Regression analysis of protein components, hay yield, and P application showed that PA, PB_1_, PB_2_, PB_3_, PC and hay yield showed a parabolic relationship with P application under low or high P rates. Under low N conditions, PA, PB_3_ and hay yield contents showed significant positive correlations with phosphorus rates, and the coefficient of determination values (R^2^) were 0.47, 0.39 and 0.63, respectively. However, PB_2_ and PC contents showed significant and negative correlations, and the coefficient determination values (R^2^) were 0.45 and 0.76, respectively (**Figure 5**). Under high N condition, PA and hay yield contents showed significant and positive correlations with phosphorus rates and the coefficient determination values (R^2^) were 0.45 and 0.59, respectively. However, PB_2_ contents showed significant and negative correlations, and the coefficient of determination values (R^2^) was 0.49 (**Figure 6**).

### 3.6 Principal component analysis and comprehensive evaluation

**Figure 7:**
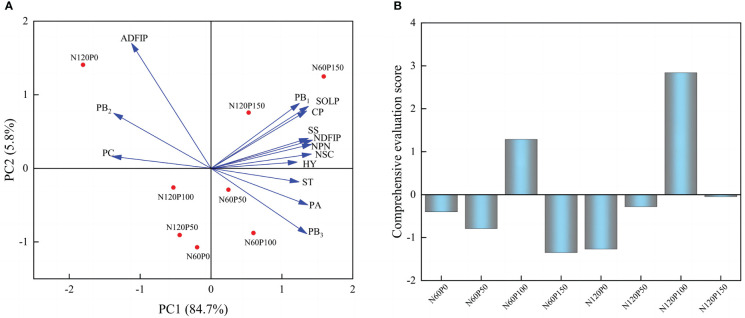
Principal component analysis of protein fractions and non-structural carbohydrates of alfalfa. **(A)** Principal component analysis, **(B)** Comprehensive evaluation score.


To investigate the effect of different N and P combined fertilization strategies on the hay yield and quality of alfalfa at the first flowering stage, we assessed the response of NSC, nitrogen distribution and protein fractions on the hay yield of alfalfa by principal coordinate analysis (PCA). Extracting the principal component eigenvalues greater than 1, two principal components were obtained (**Table 1**), it was found that the first and second axes explained 90.5% of the total variation (**Figure 7A**). The results of the analysis can be used to replace the original fourteen indicators with two principal component variables, PCA1 and PCA2, yielding the following eigenvectors for each principal component:

**Table 1:** Principal component score coefficient matrix.

Index	PC1	PC2
CP (Crude protein)	0.270	0.293
SOLP (Soluble protein)	0.275	0.317
NDFIP (Neutral detergent-insoluble protein)	0.286	0.144
ADFIP (Acid detergent-insoluble protein)	-0.224	0.637
NPN (Non-protein nitrogen)	0.283	0.122
PA (Fraction A)	0.273	-0.186
PB_1_ (Fraction B_1_)	0.249	0.331
PB_2_ (Fraction B_2_)	-0.274	0.281
PB_3_ (Fraction B_3_)	0.270	-0.333
PC (Fraction C)	-0.279	0.061
SS (Soluble sugar)	0.274	0.153
ST (Starch)	0.249	-0.069
NSC (Non-structural carbohydrate)	0.283	0.073
HY (Hay yield)	0.243	0.032

$$\begin{array}{c} PC1 = 0.270 MX1 + 0.275 MX2 + 0.286 MX3 – 0.224MX4 + 0.283 MX5 + 0.273 MX6+\\ 0.249 MX7 – 0.274 MX8 + 0.270 MX9 – 0.279 MX10+\\ 0.274 MX11 + 0.249 MX12 + 0.283 MX13 + 0.243 MX14 \end{array}$$

$$\begin{array}{c} PC2 = 0.293 MX1 + 0.317 MX2 + 0.144 MX3 + 0.637 MX4+\\ 0.122 MX5 – 0.186 MX6 + 0.331 MX7 + 0.281 MX8 – 0.333 MX9 + 0.061MX10+\\ 0.153 MX11 – 0.069 MX12+ 0.073 MX13 + 0.032 MX14 \end{array}$$

Note: PC1 and PC2 denote the scores of principal components 1 and 2, respectively. MX1 represents CP, MX2 represents SOLP, MX3 represents NDFIP, MX4 represents ADFIP, MX5 represents NPN, MX6 represents PA, MX7 represents PB_1_, MX8 represents PB_2_, MX9 represents PB_3_, MX10 represents PC, MX11 represents SS, MX12 represents ST, MX13 represents NSC and MX14 represents HY.

A comprehensive evaluation model was constructed by using the selected variance contributions of the 1st and 2nd principal components A1 (84.7%) and A2 (5.8%) (**Figure 7A**) as weights: PC3=A1PC1 + A2PC2.

The principal component analysis revealed that the highest overall score was obtained under the treatment of 100 phosphorus application at 60 or 120 kg N hm^-2^ (**Table 2** and **Figure 7B**). Therefore, 120 kg N ha^-1^ coupled with 100 kg P ha^-1^ showed the most promising effects in terms of achieving optimal forage yield consistent with enhanced forage nutritive values.

**Table 2:** Principal components, comprehensive scores and rankings under nitrogen and phosphorus combined application.

Treatment	Principal component 1 (PC1)		Principal component 2 (PC2)	Comprehensive score (PC3)	Rank
N60	P0	-1.8036	1.40531	-0.39829	5
	P50	-0.52905	-0.2602	-0.78925	6
	P100	0.53214	0.75711	1.28925	2
	P150	-0.44164	-0.90612	-1.34776	7
N120	P0	-0.194	-1.07285	-1.26685	8
	P50	0.59907	-0.87913	-0.28006	4
	P100	1.5912	1.24684	2.83804	1
	P150	0.24588	-0.29096	-0.04508	3

PC3 denotes the composite score and A is the weight of each principal component.

## 4 Discussion

### 4.1 Effects of nitrogen and phosphorus combined application on hay yield

As a perennial legume forage, alfalfa is famous for its high quality and yield, and its hay yield is a representative indicator of production performance. The available literature is inconsistent regarding the effects of fertilization on legume forage yields, particularly in alfalfa. Some studies have found no benefit from nitrogen fertilization in legume crops, as there was no significant increase in biomass yield or quality (Xie et al., 2015). Appropriate N application has been reported to regulate the distribution of photoassimilates in the aboveground parts of plants, thereby increasing the yield and resource use efficiency of alfalfa (Zhang et al., 2021). Therefore, the relationship between N application, resource use efficiency, and biomass differs between nitrogen-fixing plants and those dependent only on mineral nitrogen. There is a certain interaction between N and P fertilization. When a threshold of nitrogen is reached, applying a certain amount of P fertilizer can again improve the yield and quality of forage. Studies have shown that N and P fertilization could shift some ecosystems from N to P limitation or N and P co-limitation (Elser et al., 2010). The results of this study showed that at 60 or 120 kg N ha^-1^, the perceived third-order polynomial relationship between alfalfa yield and P fertilization was applied. When the P application is too high, the hay yield is inhibited, and there is a positive correlation between hay yield and P application. PCA showed that the highest combined scores for hay yield, agronomic traits, protein fraction, and nonstructural carbohydrates were obtained for alfalfa at 120 kg N ha^-1^ and 100 kg P ha^-1^. Meanwhile, correlation analysis showed that SS, ST, and NSC were significantly positively correlated with hay yield. Based on these results, we assumed that there is a certain threshold for fertilization uptake by alfalfa below which fertilization can promote growth and development, whereas fertilization above the maximum uptake will negatively affect growth and development and reduce forage yield, as confirmed by previous studies (Fan et al., 2016; Zhang et al., 2021). Therefore, a rational N and P combined fertilization strategy increases the hay yield of alfalfa and improves its nutritional quality.

### 4.2 Effects of nitrogen and phosphorus combined application on non-structural carbohydrates

Carbohydrate partitioning plays an important role in plant productivity and can vary with specific life forms. Carbohydrates in plants are both structural and nonstructural. Structural carbohydrates are polysaccharides, which are components of the cell wall and provide structural support to the plant (Vorwerk et al., 2004), whereas NSC act as mediators of metabolism, energy transport, and storage in plants. Total nonstructural carbohydrates (NSC) is commonly used as an indicator of quality in forage crops (Jensen et al., 2014). Among them, NSC consist mainly of soluble sugar (SS) and starch (ST), which are reserves in plant tissues (Ishimaru et al., 2004). The total NSC concentration decreased slightly with increasing N application, whereas ST decreased with N addition (Pan et al., 2011). In contrast, plant NSC content increases with the amount of N fertilization (Liu et al., 2016), and either too low or too high N fertilization causes a decrease in ST and NSC content of rice at the tasseling stage (Cao et al., 2020). It was also found that the concentration of NSC decreased slightly with increasing N fertilization, whereas the concentration of ST decreased with the application of foliar urea (Li et al., 2018). However, in this study, it was found that ST and SS increased slightly with increasing N application under the same P treatment, whereas NSC increased significantly. This may be due to the low demand for N fertilization in alfalfa at the first flowering stage and the relatively low consumption of carbohydrates for carbon skeleton and energy by N assimilation (Cheng and Fuchigami, 2002). Therefore, N supply can increase the level of leaf NSC. P is a key element in many plant functions, including carbohydrate metabolism (Vance et al., 2003) and transport (Paponov et al., 2020). Lower P application reduces ST accumulation in flowers and young fruits, which in turn reduces plant NSC content (Hermans et al., 2006). In contrast, the transport of NSC from nutrient organs to reproductive organs under phosphorus-free conditions is greater than that under high P conditions, and the effect of nitrogen on NSC transport is related to P levels (Yan et al., 2018). Interestingly, a mixture of N and P fertilization significantly increased post-flowering leaf SS concentration in oilseed flax, and a relatively high N and P mixture significantly increased post-flowering leaf ST concentration and content. However, the high N and P treatments had the lowest pre-flowering leaf ST concentration and content (Yan et al., 2018). This is consistent with the results of this study, as SS and ST content showed a trend of increasing and then decreasing with the increase of N fertilization. Once the fertilizer application is too high, it will limit the benefits of N and P fertilization to alfalfa, reducing SS, ST, and NSC content. This study also found that SS and NSC contents tended to increase and then decrease with increasing P application under the same N fertilization treatment, whereas ST slightly increased. The accumulation of ST in P-limited would support the proposal that a phosphate-dependent translocator regulates the movement of triosephosphates out of the chloroplast. Thus, when phosphate is limiting, triosephosphates accumulate in the bundle sheath chloroplasts, resulting in a higher PGA/Pi ratio, with a corresponding activation of ADP glucose pyrophosphorylase, resulting in the accumulation of ST (Yan et al., 2018). We hypothesized that a reasonable N and P combined application strategy could help increase the accumulation of SS and ST in alfalfa, and thus increase its hay yield.

### 4.3 Effects of nitrogen and phosphorus combined application on nitrogen distribution and protein fractions

Nitrogen compounds comprise 40–50% of the protoplasm dry matter and are a constituent of amino acids, the building blocks of proteins (Rosenblueth et al., 2018). In addition, SOLP and favorable growth conditions are improved by N supply (Sugiyama et al., 1984). Previous studies have shown that soluble protein and NPN content increased with increasing levels of N fertilization (Alharbi et al., 2022). In our study, NPN and SOLP content increased with increasing N content at the same P fertilization. This is because the N fertilization levels increased the accumulation of nitrate in plants taken up by plants and reduced to ammonia, a substrate for amino acid and protein synthesis, increasing plant NPN content (Gomes et al., 2018). Among the N treatments, 120 kg N ha^-1^ was the best nutrient quantity for forage. In this study, CP and SOLP contents had a parabolic relationship with increasing P application, and their contents decreased at the highest P application rate. This may be due to the high P treatment had a stressful effect on alfalfa, reducing its nutritional value. A high mineral N rate restricts root system nodule development and decreases 120 kg N ha^-1^ fixation in legumes (Xie et al., 2015), thus limiting nitrogen compound synthesis.

The CNCPS protein fraction system visually reflects the utilization of each protein fraction by forage in the animal’s rumen (Fox et al., 1995). True proteins are extensively degraded in the rumen, contribute to the N supply of rumen microorganisms, and are incorporated into the carbon skeleton (Sniffen et al., 1992). However, the easily degradable protein of this fraction can lead to peptide construction and escape into the intestine, as the use of these fraction limits protein degradation. Previous research has indicated that increased levels of N fertilization increase nitrate accumulation in plants, which is a portion of fraction A (Johnson et al., 2001). Our results showed that the content of fractions A, B_1_, and B_3_ increased with increasing N fertilization, probably because N fertilization increased nitrogen compounds in tissues and protein synthesis in plants (Johnson et al., 2001). Fractions B_1_ and B_3_ tended to increase and then decrease with the increase in P application, probably because the addition of P promoted the absorption and utilization of N fertilization by alfalfa, which in turn increased the NPN content in the plant. (Klabi et al., 2018). However, rapid rumen proteolysis of this fraction can lead to peptide construction and escape into the intestine, as the use of these components is limiting to protein degradation. Animal performance depends on the production of microbial proteins, which can be optimized with larger amounts of soluble N. However, high concentrations of fraction A and B_1_ are desirable, as this fraction is rapidly degraded in the rumen, thereby affecting rumen homeostasis and possibly the ruminant growth performance. Fraction C corresponds to N linked to lignin, tannin-protein complexes, and merad products, which are highly resistant to enzymes produced by microorganisms in the rumen and are considered unusable for animals (Sniffen et al., 1992). In our study, it was found that fraction C decreased with an increase in N fertilization level, which was consistent with the results of previous studies (Alharbi et al., 2022). However, the fraction C content showed a trend of increasing and then decreasing with an increase in P application because P fertilization decreased the acid detergent fiber content in alfalfa, which in turn decreased the fraction C content (Zhang et al., 2014). Therefore, a good N and P combined application strategy can increase the content of fractions A and B and decrease that of fraction C, and increase the protein effectiveness of alfalfa, which in turn will benefit the growth and development of ruminants.

## 5 Conclusion

The results of the present study showed that a good N and P combined fertilization strategy could increase the soluble nitrogen compounds and total carbohydrate content of perennial alfalfa. On the other hand, reasonable P fertilization levels were more beneficial for increasing the true protein content and reducing the non-degradable protein, slowly degrading protein content, and crude fiber content of alfalfa. Therefore, the fertilization of 120 kg N ha^-1^ and 100 kg P ha^-1^ to alfalfa for consecutive years promoted its growth and development, increased its protein content, and avoided the accumulation of more structural carbohydrates, thus increasing the hay yield and nutritional quality of alfalfa.

## Data availability statement

The original contributions presented in the study are included in the article/supplementary material. Further inquiries can be directed to the corresponding authors.

## Author contributions

Conceptualisation, QZ, XW and CM. Software, ML. Validation, JZ and RH. Formal analysis, JZ. Data curation, QZ and JZ. Writing—original draft preparation, JZ. Visualisation, JZ. Supervision, QZ. Funding acquisition, QZ. All authors contributed to the article and approved the submitted version.
